# What Links Chronic Kidney Disease and Ischemic Cardiomyopathy? A Comprehensive Bioinformatic Analysis Utilizing Bulk and Single-Cell RNA Sequencing Data with Machine Learning

**DOI:** 10.3390/life13112215

**Published:** 2023-11-16

**Authors:** Lingzhi Yang, Yunwei Chen, Wei Huang

**Affiliations:** 1 Department of Cardiology, the First Affiliated Hospital of Chongqing Medical University, Chongqing 400016, China; yang_lingzhi@163.com; 2 Department of Cardiology, Nanjing First Hospital, Nanjing Medical University, Nanjing 210006, China; 18982694019@163.com

**Keywords:** ischemic cardiomyopathy, chronic kidney disease, fibroblast, scRNA-seq, machine learning

## Abstract

Chronic kidney disease (CKD) emerges as a substantial contributor to various cardiovascular disorders, including ischemic cardiomyopathy (ICM). However, the underlying molecular mechanisms linking CKD and ICM remain elusive. Our study aims to unravel these connections by integrating publicly available bulk and single-cell RNA sequencing (scRNA-seq) data. Expression profiles from two ICM datasets obtained from heart tissue and one CKD with Peripheral Blood Mononuclear Cell (CKD-PBMC) dataset were collected. We initiated by identifying shared differentially expressed genes (DEGs) between ICM and CKD. Subsequent functional enrichment analysis shed light on the mechanisms connecting CKD to ICM. Machine learning algorithms enabled the identification of 13 candidate genes, including AGRN, COL16A1, COL1A2, FAP, FRZB, GPX3, ITIH5, NFASC, PTN, SLC38A1, STARD7, THBS2, and VPS35. Their expression patterns in ICM were investigated via scRNA-seq data analysis. Notably, most of them were enriched in fibroblasts. COL16A1, COL1A2, PTN, and FAP were enriched in scar-formation fibroblasts, while GPX3 and THBS2 showed enrichment in angiogenesis fibroblasts. A Gaussian naïve Bayes model was developed for diagnosing CKD-related ICM, bolstered by SHapley Additive exPlanations interpretability and validated internally and externally. In conclusion, our investigation unveils the extracellular matrix’s role in CKD and ICM interplay, identifies 13 candidate genes, and showcases their expression patterns in ICM. We also constructed a diagnostic model using 13 gene features and presented an innovative approach for managing CKD-related ICM through serum-based diagnostic strategies.

## 1. Introduction

Chronic kidney disease (CKD) is a significant global health concern, impacting over 13.4% of the world’s population, and its prevalence continues to rise [1]. The development of CKD involves an intricate interplay of factors like inflammation, oxidative stress, and metabolic dysregulation, which not only contribute to progressive kidney function decline but also increase the risk of other conditions, including ischemic cardiomyopathy (ICM) [2]. CKD is considered one of the strongest risk factors for developing cardiovascular disease, to which coronary artery disease contributes the large part of adverse events [3]. Numerous studies have revealed coronary artery narrowing and occlusion [4], along with a high prevalence of myocardial ischemia and heart dysfunction in CKD patients [5,6]. However, the precise molecular mechanisms connecting these two diseases are not fully revealed.

Studies have indicated that an abundance of internal and external mediators can trigger systemic, chronic inflammatory state in CKD, which could potentially contribute to various cardiovascular diseases [6]. Additionally, studies have shown that CKD mimicked accelerated aging [7], evidenced by the accumulation of senescent cells [8] and increased levels of inflammatory markers of the senescence-associated phenotype [9]. The cellular senescence could contribute to atherosclerosis [10], calcification and cardiac remodeling following ischemic events [11]. All of these findings suggest the potential involvement of secretory proteins in CKD-related ICM. In addition, due to the high incidence of coronary artery disease in individuals with CKD [12] and the limitations of current imaging modalities [13], it is crucial to identify those with a higher risk of myocardial ischemia based on plasma biomarkers.

In this study, we used public datasets for reanalysis to identify commonly regulated genes in ICM and CKD, aiming to unravel potential mechanisms underlying CKD-related ICM. Through a machine-learning feature selection method, we identified 13 potential candidate genes encoding secretory proteins. Subsequently, we investigated the expression pattern of these candidate genes in ICM using single-cell RNA sequencing (scRNA-seq) data. Finally, we constructed a diagnostic model based on candidate genes using machine-learning algorithms and validated its performance internally and externally.

## 2. Methods

### 2.1. Data Collection

**Figure 1:**
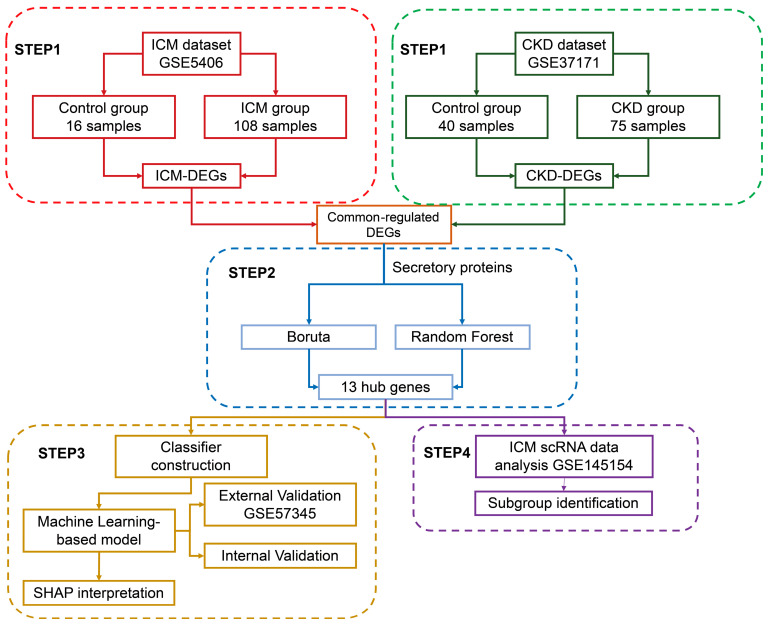
Flowchart of current study. ICM, ischemic cardiomyopathy. CKD, chronic kidney disease. DEG, differentially expressed gene. SHAP, SHapley Additive exPlanations.

For the current study, we utilized publicly available datasets of ICM and CKD. GSE5406, GSE57345 were selected for ICM, which contain microarray data from cardiac tissue samples obtained from individuals with ICM. In our study, we employed GSE5406 for conducting differential analysis and model construction, while GSE57345 served as the dataset for external validation. The GSE37171 dataset, encompassing microarray data acquired from peripheral blood mononuclear cell (PBMC) samples taken from CKD patients, was also included in our analysis. We incorporated the GSE145154 dataset, which includes scRNA-seq data from cardiac tissue samples of individuals with ICM, in order to explore cellular heterogeneity and identify gene expression patterns in ICM. Table 1 provides the details of the included dataset, while Figure 1 visualizes the workflow of the current study.

### 2.2. Differentially Expressed Genes (DEGs) Analysis

In this study, we employed the “limma” package [14] within the R software (https://www.r-project.org/) to identify Differentially Expressed Genes (DEGs) in datasets related to ICM and CKD. DEGs in both the ICM and CKD datasets were filtered using stringent criteria, specifically, an adjusted *p*-value ≤ 0.05 and an absolute value of log2 (fold change) ≥ 0.25. To identify commonly regulated genes in both ICM and CKD datasets, we focused on genes that exhibited consistent upregulation or downregulation in both diseases. For visualization, we employed the “ggplot2” [15] and “Complexheatmap” [16] packages within the R software. The expression patterns of DEGs were presented through dot plots and heatmaps, respectively, enabling a comprehensive visual analysis of their expression levels and regulation changes.

### 2.3. Functional Enrichment Analysis

To gain insight into the biological functions and underlying mechanisms of DEGs, gene oncology (GO) term (http://geneontology.org/docs/gocitation-policy/) analysis was conducted via the “clusterprofiler” [17] package of R. We utilized the “fgsea” package [18] to conduct Gene Set Enrichment Analysis (GSEA) using KEGG pathway gene sets sourced from the “msigdb” database.

### 2.4. Protein–Protein Interaction (PPI) Network Construction

To investigate the associations among common DEGs in the two diseases, we established a protein–protein interaction (PPI) network based on data from the STRING database (https://www.string-db.org) on the basis of physical interactions, coexpression, and curated knowledge, with a confidence score of at least 0.4. We then visualized the PPI network and calculated the node degree to show intra-network connectivity using Cytoscape software (version 3.8.2).

### 2.5. Secretory Proteins Access

In this study, secretory proteins were obtained from the Human Protein Atlas database (https://www.proteinatlas.org/). Specifically, a total of 3947 genes coding for secretory proteins were downloaded from the protein class labeled as “SPOCTOPUS predicted secreted proteins”.

### 2.6. Feature Selection Based on Machine Learning

Machine learning methodologies were employed to discern potential candidate genes associated with CKD-related ischemic cardiomyopathy (ICM). Specifically, we harnessed the Least Absolute Shrinkage and Selection Operator (LASSO) algorithm, Boruta algorithm and the Random Forest (RF) algorithm to perform the task of feature selection. The LASSO algorithm stands out for its simplicity, quantitative feature importance, and applicability to high-dimensional data. The Boruta algorithm is a feature selection technique that builds upon the RF algorithm, enhancing its capabilities. RF, on the other hand, leverages ensemble learning principles by aggregating multiple decision trees to improve prediction accuracy. In our study, the “glmnet” package [19], “Boruta” package [20] and “randomForest” package [21] were utilized for this purpose. We utilized cross-validation to determine the optimal regularization lambda for the LASSO algorithm. In the case of the Boruta algorithm, we configured its parameters as follows: the significance level was set to 0.05 (*p* Value = 0.05), the Monte Carlo adjustment was enabled (mcAdj = T), and a maximum of 300 runs were conducted (maxRuns = 300). We maintained the default values for all the other parameters. For the RF algorithm, we adhered to the default settings and selected features with a Mean Decrease in Accuracy greater than 4. The overlapping genes of three feature selection algorithms were selected for further analysis.

### 2.7. scRNA-Seq Data Analysis

In this study, we conducted an analysis of public scRNA-seq data from GSE145154 to investigate the candidate genes’ expression patterns in ICM. Firstly, we preprocessed the data and the filtering criteria for scRNA-seq data as follows: cells should have more than 200 but less than 10,000 detected RNA features in order to exclude low-quality or excessively abundant cells, and the percentage of mitochondrial genes (pMT) in each cell should be less than 5% to remove damaged or dying cells. Then, initial count matrix was subjected to normalization and scaling using the “SCTransform” method [22]. Principal component (PC) analysis was conducted using the Seurat package as a technique for dimensional reduction, leveraging the variable genes. The determination of the appropriate number of PCs involved the utilization of both an elbow plot and a quantitative approach. The inflection point was carefully identified by the following criteria: (1) the individual principal components contributed only 5% of the standard deviation; (2) their cumulative effect accounted for 90% of the standard deviation; and (3) the point was pinpointed at which the percent change in variation between consecutive principal components fell below 0.1%. Next, the batch effect was corrected using the harmony approach [23]. Subsequently, the identified PCs were employed to construct a shared nearest neighbor graph, which was then subjected to clustering using the Louvain method, adopting a resolution of 0.2. To further reduce dimensions, the uniform manifold approximation and projection (UMAP) technique [24] was applied. To identify genes specific to particular cell clusters, we employed the Seurat package and considered genes displaying significant differences in activity between clusters, setting a threshold of Log2FC greater than 0.25 to pinpoint these DEGs using the “FindAllMarker” function from the Seurat package [25]. For cell type annotation, we manually curated the data, referring to established resources like PanglaoDB [26] and CellMarker 2.0 [27]. For the annotation of subclusters of fibroblast, we referred to previous literature [28].

### 2.8. Classifier Construction and Assessment Based on Machine Learning Algorithm

The ICM dataset GSE5406 was randomly split into a random 70–30 division, with 70% of the data used for training and the remaining 30% for model testing. To create classifiers, we employed four machine learning algorithms, combining GaussianNB (Gaussian Naive Bayes), XGBClassifier (eXtreme Gradient Boosting), RandomForestClassifier, and KNeighborsClassifier (K-Nearest Neighbors). We employed GridSearch [29], which is a technique to systematically explore different hyper-parameter settings for our machine learning model. We then used the best-performing parameters for further analysis (Supplementary Information, Table S1). For a comprehensive assessment of classifier performance, a range of metrics, including the Brier Score, precision, recall, F1 score, and AUC for the ROC curve, were employed. Subsequently, the classifier with the best performance in the test set was chosen. Additionally, we utilized a calibration curve to gauge the alignment between predictions and observations. In order to comprehend and interpret the gene features derived from the classifier model, the SHapley Additive exPlanations (SHAP) method [30] was employed. This method allowed for an analysis of feature attribution, enabling a better understanding of the results produced by the machine learning-based classifier. The Jupyter notebook was used for analysis and results visualization. The dataset GSE57345 was used for external validation of the model performance.

### 2.9. Statistical Analysis

The R software and Jupyter notebook were employed for conducting statistical analyses and data visualization. In order to gauge distinctions between the two groups, the Wilcoxon rank-sum test was utilized.

## 3. Results

### 3.1. Identification of DEGs in ICM and CKD and Functional Enrichment Analysis

**Figure 2:**
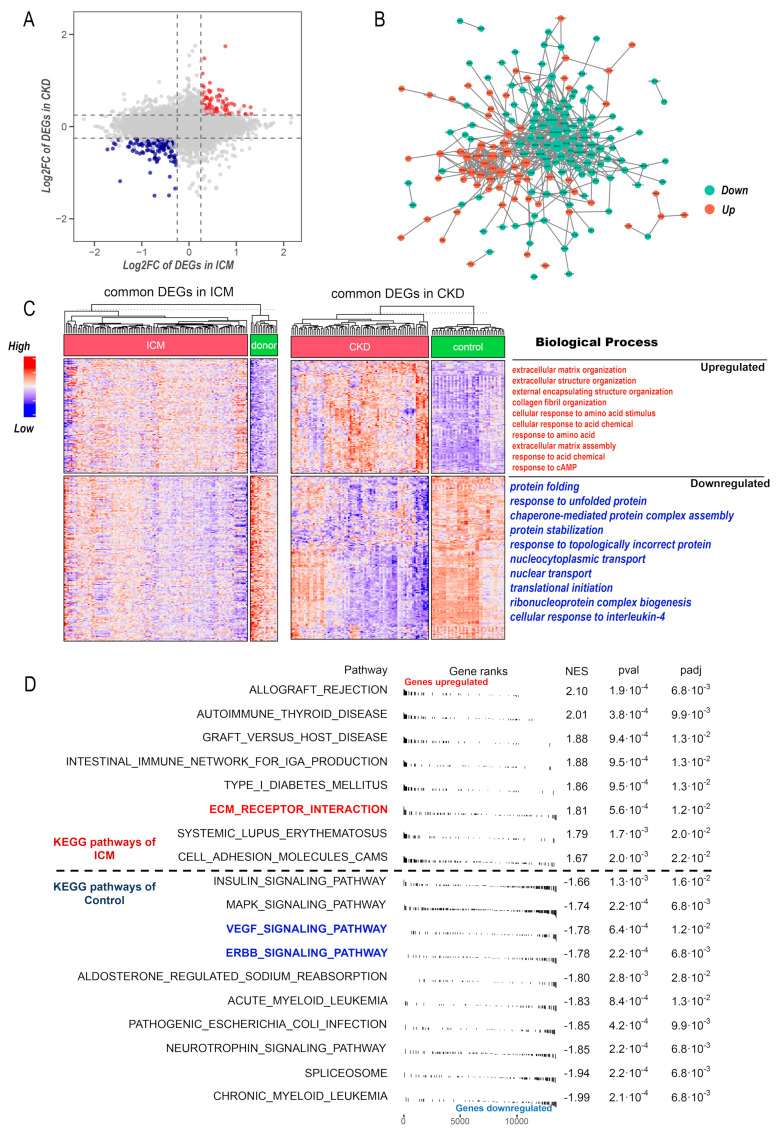
Identification of commonly regulated genes between ICM and CKD. (**A**) Dotplot illustrating the expression changes of DEGs in ICM and CKD. The x-axis represents the log2FC of DEGs in ICM, while the y-axis represents the log2FC of DEGs in CKD. Each dot on the plot corresponds to a gene, with its position indicating the magnitude of its regulation in both ICM and CKD. (**B**) PPI network of commonly regulated genes in the ICM and CKD, the size of node shows the degree of intra-connectivity. (**C**) heatmap showing the expression pattern of commonly regulated genes in ICM (**left**) and CKD (**right**) datasets. Biological process annotation of these genes is shown on the right. (**D**) GSEA results in the context of gene sets from KEGG pathway database in ICM dataset from GSE5406.

By conducting differential analysis in ICM and CKD datasets separately, we identified 264 DEGs with consistent regulation in two diseases. Among these DEGs, 108 genes displayed upregulation, while 156 genes exhibited downregulation (Figure 2A). The protein–protein (PPI) network was constructed based on STRING database and node degree was calculated (Figure 2B). Subsequently, we performed a GO term enrichment analysis on the commonly regulated genes in both diseases. The heatmap of common DEGs and top enriched GO terms are illustrated in Figure 2C. Noticeably, the GO term enrichment analysis highlighted biological processes associated with the construction of the extracellular matrix (ECM), encompassing ECM organization, ECM assembly, and collagen fibril organization. GSEA results unveiled that ECM related pathway was activated while Erythroblastic Oncogene B (ERBB) and vascular endothelial growth factor (VEGF) signaling pathways were downregulated in ICM (Figure 2D).

### 3.2. Identification of Candidate Genes for CKD-Related ICM

**Figure 3:**
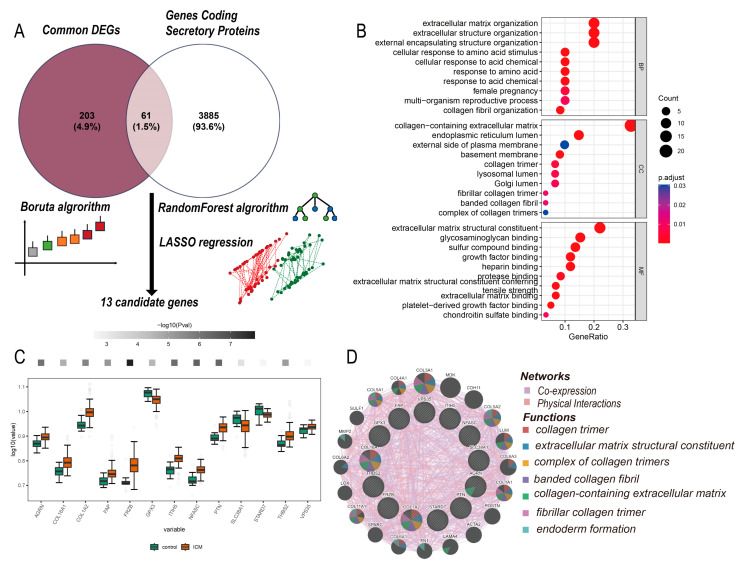
(**A**) Candidate genes selection. The commonly regulated genes between ICM and CKD were intersected with secretory proteins first. Then, LASSO, Boruta and Random Forest algorithms were used for filtering features. (**B**) Dotplot showing GO term enrichment annotations for commonly regulated secretory protein-encoding genes. (**C**) Boxplot showing expression of candidate genes in ICM and control group. (**D**) A network showing coexpression and physical interactions of candidate genes using GeneMANIA.

Considering that secretory proteins are involved in ECM composition and the potential implication of secretory proteins to CKD-related ICM, we intersected genes encoding secretory proteins with common DEGs and obtained 61 genes (Figure 3A). The enrichment analysis of these 61 genes informed that they were involved in extracellular matrix construction and mainly located in collagen-containing extracellular matrix (Figure 3B). In order to identify candidate genes among the 61 candidate ones, we then combined LASSO, Boruta and RF algorithms for feature filtering. The LASSO algorithm identified 14 DEGs with coefficients exceeding one standard error as candidate genes. 25 DEGs were identified as the candidate genes of ICM by Boruta algorithm and genes with MeanDecreaseAccuracy > 4 from RF model were extracted as candidate genes (Supplementary Information, Figures S1–S3). Thirteen genes were screened out after the three intersected. The expression levels of these 13 genes in ICM and control group are shown in Figure 3C. We also showed the coexpression and physical interactions of the candidate genes using the GeneMANIA [31] online tool (Figure 3D). The complete network is shown in Supplementary Information, Figure S4.

### 3.3. scRNA-Sequencing Analysis of ICM

**Figure 4:**
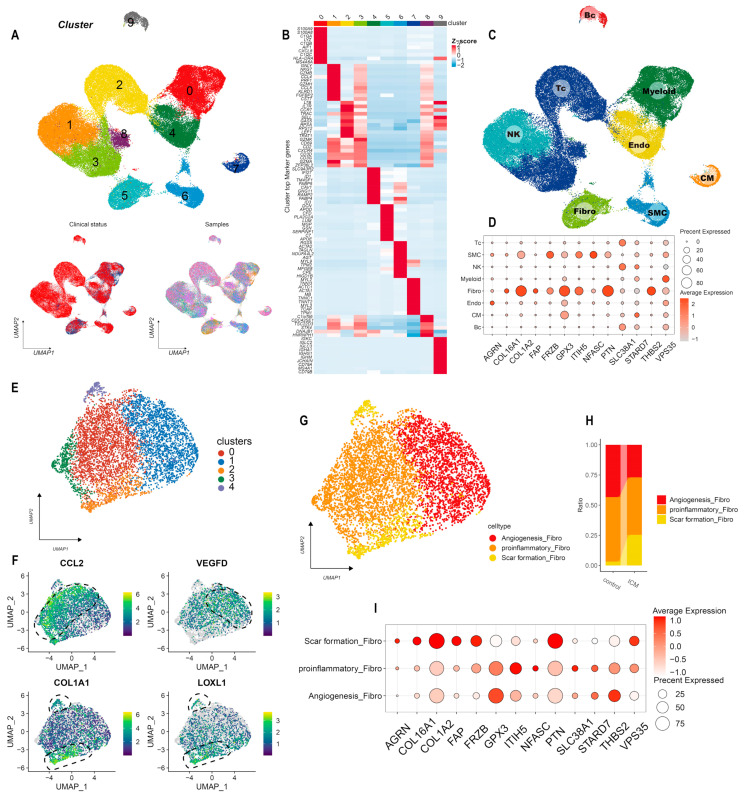
(**A**) UMAP embedding of scRNA-seq data from human heart. (**B**) Heat map displays markers for each cluster. (**C**) Annotated UMAP plot for human heart. (**D**). Bubble plot showing candidate genes expression in each type of cell. (**E**) UMAP embedding of fibroblast subclusters. (**F**) Feature plots depict the expression of the CCL2, VEGFD, COLA1, and LOXL1 within a UMAP plot. (**G**) Annotated UMAP plot for fibroblasts. (**H**) Proportional distribution of cell types in different group. (**I**) Bubble plot showcases the expression patterns of candidate genes across diverse fibroblast subclusters.

To further identify the expression pattern of candidate genes in ICM, we reanalyzed public dataset GSE145154 from the heart tissue of ICM and donor samples. We used UMAP to decrease the dimensionality of the scRNA expression data. The UMAP plot displayed a significant overlap of all samples, indicating successful integration and representation of cell populations from both the donor and ICM groups (Figure 4A). The top markers of each cluster are shown in Figure 4B. Through manual annotation, we identified eight distinct cell types (Figure 4C). We found most of the candidate genes were highly expressed within the fibroblast population (Figure 4D). Considering the substantial enrichment of candidate genes within fibroblasts, which play a pivotal role in ECM construction [32], we further classified fibroblasts into subclusters (Figure 4E). A previous study indicated that post-myocardial infarction secreted CCL2 (chemokine (C-C motif) ligand 2) and developed a proinflammatory phenotype [33]. Additionally, fibroblasts are known to stimulate endothelial cells (ECs) by secreting VEGF (Vascular endothelial growth factor), promoting angiogenesis and revascularization [34]. Additionally, encoding COLA1 (Collagen Type I Alpha 1 Chain) and LOXL1 (Lysyl Oxidase Like 1) in fibroblast characterized composition of extracellular matrix for scar formation [28]. Based on the expression of specific genes, we classified the fibroblasts into 3 subclusters (CCL2 for pro-inflammation, VEGFD (Vascular Endothelial Growth Factor D) for angiogenesis, and COLA1 and LOXL1 for scar formation) (Figure 4F,G). A larger portion of cells belonged to the scar-formation fibroblast subcluster, while the angiogenesis fibroblast subcluster represented a smaller fraction (Figure 4H). We then found AGRN (Agrin), COL16A1 (Collagen Type XVI Alpha 1 Chain), COL1A2, FAP (Fibroblast Activation Protein Alpha), FRZB (Frizzled Related Protein), PTN (Pleiotrophin), and VPS35 (VPS53 Subunit of GARP Complex) are mainly expressed in scar-formation fibroblasts, while GPX3 (Glutathione Peroxidase 3) and THBS2 (Thrombospondin 2) were mainly enriched in angiogenesis fibroblasts (Figure 4I).

### 3.4. Construction and Validation of a Diagnostic Model for CKD-Related ICM Using Machine Learning Algorithms

**Figure 5:**
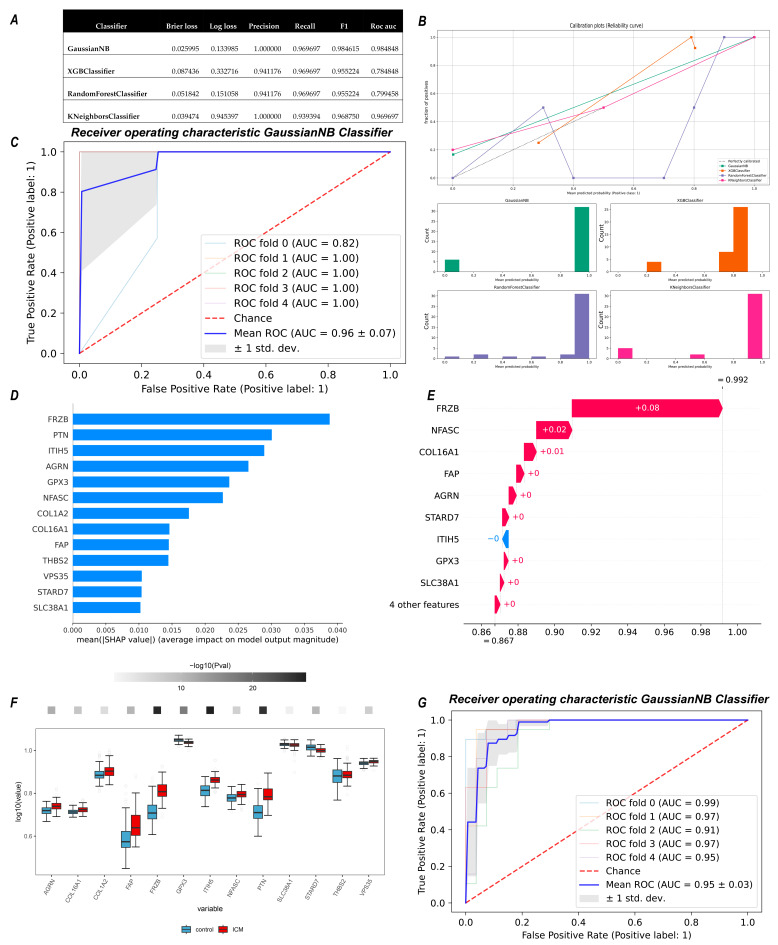
Classifier construction, validation and visualization. (**A**) Statistical parameters of four classification algorithms in train cohort. (**B**) The calibration curve of four classifiers. (**C**) ROC plot of GaussianNB algorithm-based classifier in test cohort after 5-fold cross-validation. (**D**) Importance plot of GaussianNB algorithm-based classifier. (**E**) The waterfall plot shows an example explanation on an individual case. (**F**) Boxplot showing expression of candidate genes in ICM and control group from GSE57345 dataset. (**G**) External 5-fold cross-validation of GaussianNB model in GSE57345.

To facilitate the diagnosis of ICM patients in CKD, we constructed a diagnostic model based on a panel of 13 candidate genes. Four machine-learning algorithm-based classifiers were tested. The well-tuned ensemble models displayed impressive performance in the train set, but they struggled to generalize effectively to the test set (Supplementary Information, Figure S5). However, the GausssianNB classifier showed the lowest Brier loss and the highest discrimination performance (AUC: 0.98484) (Figure 5A,B). After internal validation using the five-fold cross-validation method in the test cohort, the model yielded an AUC of 0.96 (95% confidence interval 0.89–1.00) in the test cohort (Figure 5C). We then showed the interpretation of the GaussianNB model using the SHAP method. For each prediction, a positive SHAP value indicates an increase in the predisposition to ICM and vice versa (Figure 5D). A waterfall plot shows the local interpretability of the model (Figure 5E). For external validation, we extracted data from GSE57345 and found consistent expression pattern of candidate genes (Figure 5F) and outstanding performance of the current diagnostic model in the external dataset (AUC = 0.95 ± 0.03) (Figure 5G).

## 4. Discussion

Currently, CKD is increasingly imposing burdens on global health, and its related cardiovascular diseases have become the leading risk of mortality and morbidity [3]. CKD-related ICM often presents as asymptomatic or with atypical symptoms, posing a challenge for accurate diagnosis and risk stratification. Additionally, the underlying mechanisms linking CKD and ICM remain not fully understood. By using comprehensive bioinformatic analyses, our study investigated pathogenic genes linking CKD and ICM. In both CKD and ICM patients, the genes regulating ECM formation are enriched, and secretory proteins play important roles in it. Using machine learning algorithms, we identified 13 candidate genes linking the two diseases, including AGRN, COL16A1, COL1A2, FAP, FRZB, GPX3, ITIH5 (Inter-Alpha-Trypsin Inhibitor Heavy Chain 5), NFASC (Neurofascin), PTN, SLC38A1 (Solute Carrier Family 38 Member 1), STARD7 (StAR-related lipid transfer domain protein 7), THBS2, and VPS35. By integrating scRNA-seq data analysis, we found that the candidate genes were predominantly expressed by fibroblasts. Through an in-depth analysis focusing on fibroblast subclusters, we found that the ICM group exhibited a higher proportion of cells dedicated to scar formation and a lower proportion for angiogenesis. Within scar-formation subclusters, specific genes such as COL16A1, COL1A2, PTN, and FAP were found to be remarkably enriched. Conversely, within angiogenesis-related subclusters, genes like GPX3 and THBS2 displayed substantial enrichment. Considering the challenge of diagnosing and managing CKD-related ICM, we constructed a GaussianNB algorithm-based diagnostic model to identified patients with risk of myocardial ischemia with CKD. Furthermore, we made the model interpretable by using the SHAP method and validating its outstanding performance in the external dataset.

In the context of ICM, therapeutic angiogenesis has been shown to revascularize ischemic heart tissue, reducing the progression of tissue infarction and ischemia [35]. Fibroblast is a dynamic cell type and it has been reported to participate in angiogenesis by secreting VEGF [34] and angiopoietin 1 [36]. In our study, we showed a lower level of angiogenesis fibroblasts in ICM group. Additionally, GPX3, which was downregulated in ICM group, was mainly enriched in this subcluster of fibroblasts. GPX3 serves as an extracellular glutathione peroxidase and has implications for various diseases. The loss of GPX3 resulted in kidney fibrosis through reactive oxygen species generation and p38 mitogen-activated protein kinase activation [37]. Genetic variance of GPX3 is associated with severity of coronary artery disease [38], and a previous study showed that, in the context of CKD, GPX3 deficiency could lead to the activation of platelet and result in coronary artery occlusion, left ventricular dysfunction [39]. Furthermore, a recent study presented a statistical model for predicting heart dysfunction of ICM based on GPX3 level. A single-cell analysis also reported the potential involvement of GPX3 in cardiac fibroblast differentiation under pressure overload [40]. Furthermore, the role of glutathione peroxidase in the interaction of fibroblast and endothelial cells was investigated since GPX1, another glutathione peroxidase, was reported to participate in maintaining endothelial progenitor cell function and angiogenesis [41]. The role of GPX3 in fibroblasts and CKD-related ICM warrants further investigation. THBS2 participates in ECM assembly and inhibition of angiogenesis [42]. A study has shown miR-29a-3p/THBS2 axis is involved in pulmonary artery hypertension-induced cardiac fibrosis [43]. Our study showed THBS2 significantly increased in ICM and it mainly enriched in angiogenesis fibroblast, which is consistent with previous findings. Scar formation is the main function of activated fibroblasts in cardiac remodeling [28], FAP is a prolyl-specific serine protease, and the latest work suggested that it could be a prognostic marker for ischemic injury of heart and its inhibition could promote cardiac repair by stabilizing B-type natriuretic peptide [44]. Our work showed FAP significantly increased in ICM and it is mainly expressed in scar-formation fibroblast. Its role in CKD-related ICM warrants further investigation. PTN has been reported to hold therapeutic promise in ICM owing to its capacity to enhance neovasculature formation [45], but its high expression was also reported to develop peritoneal fibrosis in chlorhexidine gluconate-induced peritoneal fibrosis mice [46]. In the current study, PTN was increased in ICM group, especially in scar-formation fibroblasts, while its specific function is still not fully understood.

There is a high prevalence of silent myocardial ischemia owing to diabetic or uremic neuropathy, especially in end-stage renal disease patients [47]. A myocardial fatty acid imaging study showed severely impaired myocardial ischemia among hemodialysis patients [48]. In addition, as kidney disease progresses, distinguishing between ICM and the symptoms of uraemia and anaemia becomes increasingly difficult since fatigue and dyspnoea are atypical and common in both conditions [12]. A previous study showed the value of tissue Doppler echocardiography [49] and magnetic resonance imaging in the estimation of heart function and prognosis of CKD [50]. Given the limitations posed by the echocardiography operator’s skills and the imaging quality, there is a requirement to discover additional conventional serum biomarkers for the diagnosis and risk stratification of CKD patients with ICM. Our study constructed a reliable diagnostic model based on 13 candidate genes encoding secretory proteins and machine learning algorithm. We utilized the SHAP method for model interpretation as well. The external validation of the current diagnostic model in another dataset performed well. The use of the current model should also be tested in clinical samples from CKD patients. In addition, we found the Naïve Bayes-based classifier showed stable and great performance while those ensemble methods did not. This could be attributed to the conditions suitable for Naive Bayes and limited data size. We suggest that future research should evaluate more up-to-date classification algorithms, including recurrent neural network in larger database. Bridging the gap between basic science and clinical practice is challenging, but with the assist of some advanced molecular phenotyping technologies [51,52], we anticipate that our model will find clinical applicability in predicting CKD-related ICM and risk stratification.

We acknowledge that the primary analyses in our study were conducted in silico, which represents a limitation of our research. While in silico analyses provide valuable initial insights, further in vivo and in vitro experiments are necessary to delve deeper into and validate our findings.

## 5. Conclusions

Our research unveiled the crucial pathways associated with the ECM that underlie the relationship between CKD and ICM. By combining scRNA-seq data, we further discovered the candidate genes mainly enriched in fibroblasts. It was found that COL16A1, COL1A2, PTN, and FAP were remarkably enriched in scar-formation fibroblasts while GPX3 and THBS2 displayed substantial enrichment within angiogenesis-related subclusters. We also showed an increased level of scar-formation fibroblasts in our study. These specific gene expression and cardiac fibroblast composition patterns suggested an inclination towards cardiac fibrosis. Furthermore, we successfully constructed a diagnostic model based on a machine-learning algorithm for ICM, which was validated internally and externally. This offers novel insights into potential serum-based diagnostic and management strategies of CKD with ICM.

## Figures and Tables

**Table 1:** Characteristics of GEO datasets.

GEO Accession	Platform	Origin	Sample		Species
			Control	Disease	
GSE5406	GPL96	Heart	16	108	Homo Sapiens
GSE37171	GPL570	PBMC	40	75	Homo Sapiens
GSE57345	GPL11532	Heart	136	95	Homo Sapiens
GSE145154	GPL20795	Heart	5	14	Homo Sapiens

## Data Availability

The public datasets were downloaded and analyzed in this study, which can be found in GEO data repository and included the accession numbers as follows: GSE5406, GSE37171, GSE57345, GSE145154.

## Supplementary Materials

The following supporting information can be downloaded at: https://www.mdpi.com/article/10.3390/life13112215/s1, Figure S1. Boxplot showing the importance of selected genes by Boruta algorithm; Figure S2. The RF algorithm presenting the MeanDecreaseAccuracy of genes in ICM and 13 biomarkers with the score more than 4.0 were selected; Figure S3. Feature genes screening in the LASSO algorithm with 14 candidate hub genes selected; Figure S4. Relationship of candidate genes; Figure S5. Results of tuning process and performance based on GridsearchCV on train (left) and test (right) set. Table S1. Hyper-parameters set for each classifier.
