# Supplementary material for: What Links Chronic Kidney Disease and Ischemic Cardiomyopathy? A Comprehensive Bioinformatic Analysis Utilizing Bulk and Single-Cell RNA Sequencing Data with Machine Learning
Source: Life (Basel). 2023 Nov 16;13(11):2215. doi: 10.3390/life13112215 (PMC10672726; doi:10.3390/life13112215)
Supplement: Supplementary file 1 [file life-13-02215-s001.zip › life-2629552-supplementary.pdf]

Supplementary Information

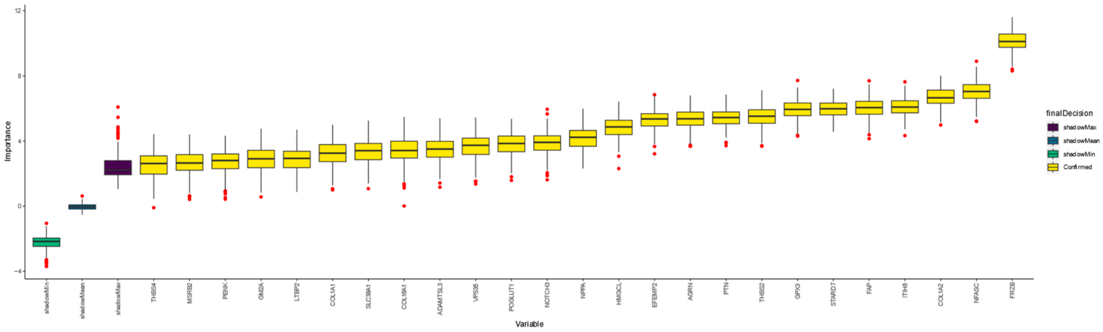

Figure S1. Boxplot showing the importance of selected genes by Boruta algorithm.

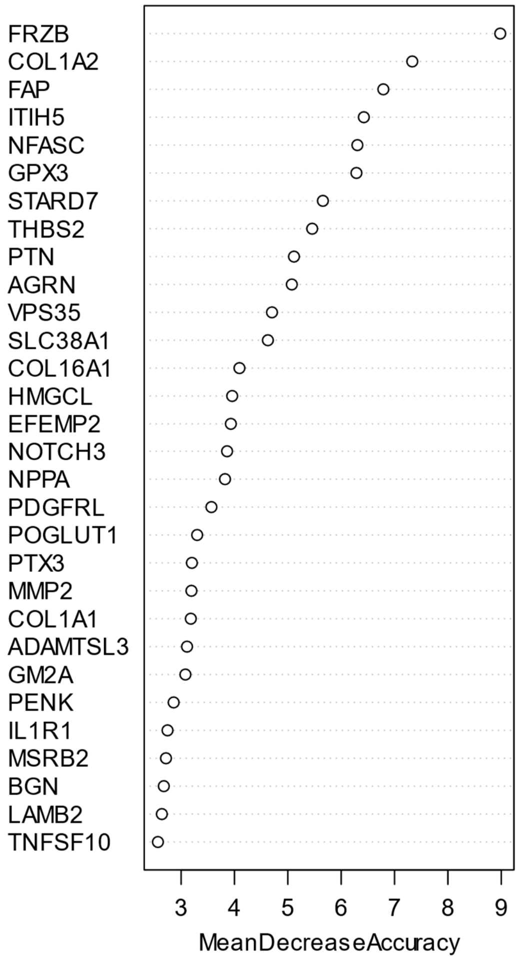

Figure S2. The RF algorithm presenting the MeanDecreaseAccuracy of genes in ICM and 13 biomarkers with the score more than 4.0 were selected.

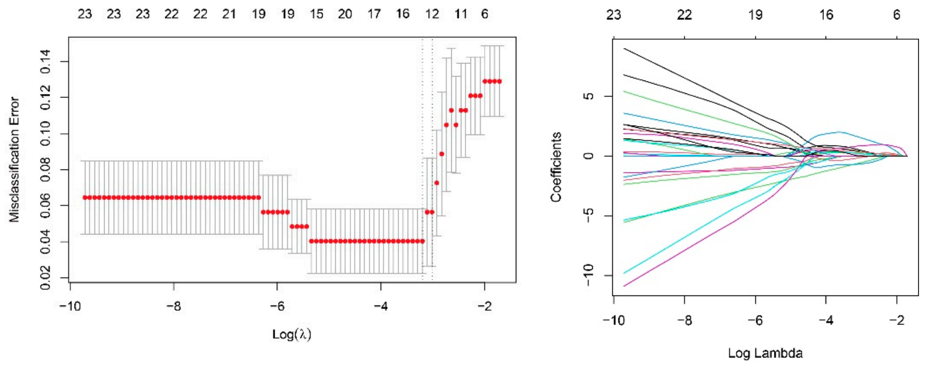

Figure S3. Feature genes screening in the LASSO algorithm with 14 candidate hub genes selected. Feature genes selected by LASSO: GPX3, ITIH5, PTN, NFASC, SLC38A1, STARD7, THBS2, VPS35, AGRN, COL16A1, COL1A2, FAP, FRZB, NOTCH3.

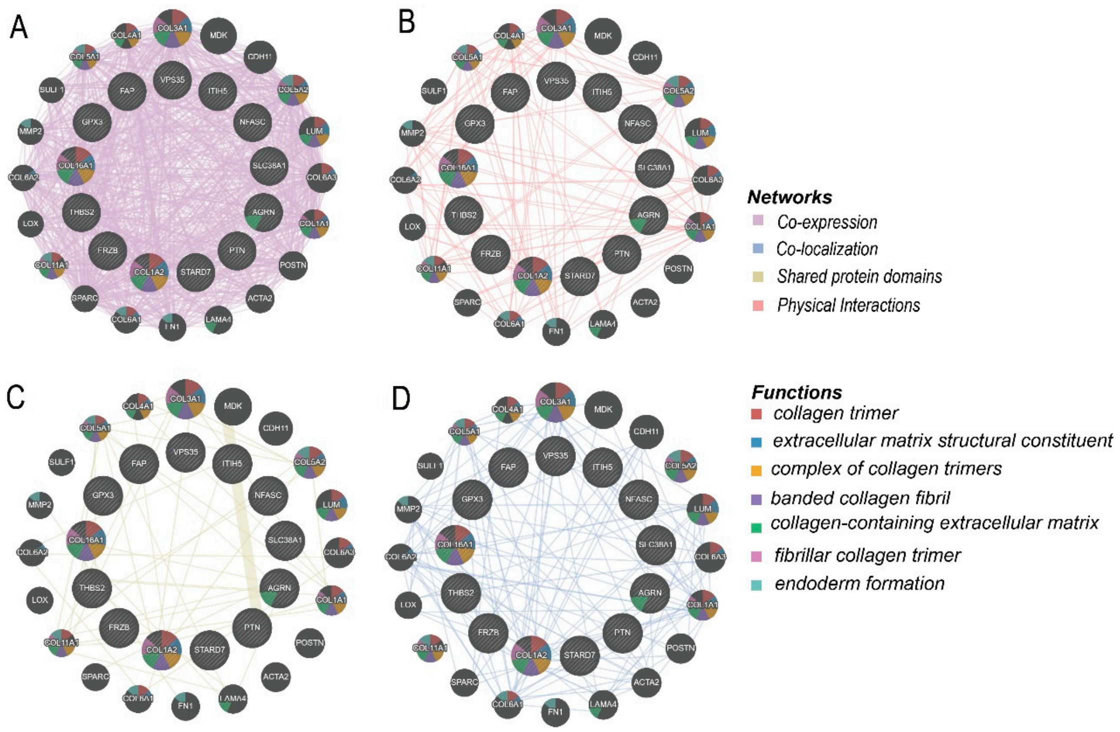

Figure S4. Relationship of candidate genes. (A) Co-expression, (B) Physical interaction, (C) Shared protein domains, (D) Co-localization relationship of candidate genes.

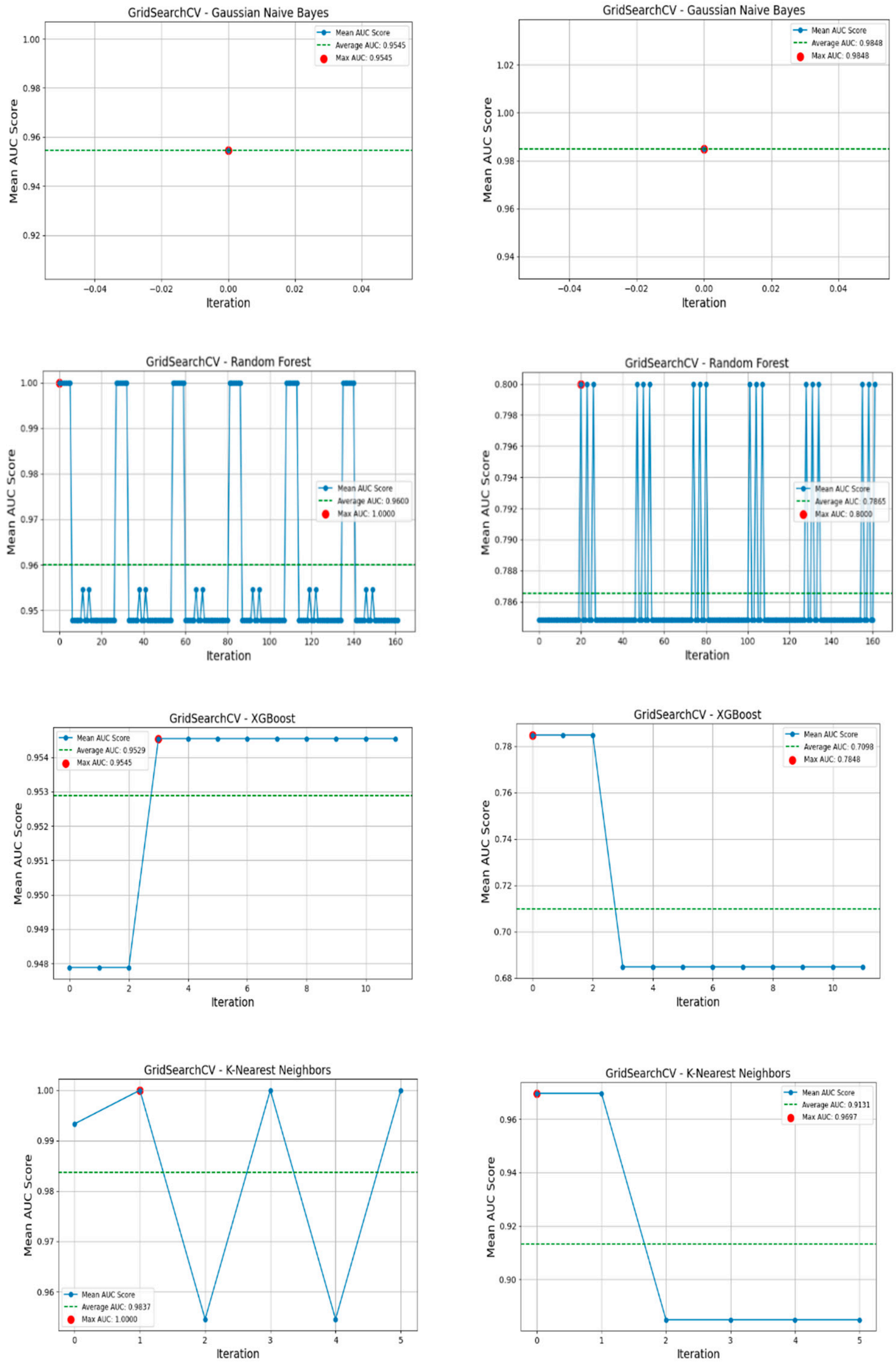

Figure S5. Results of tuning process and performance based on GridsearchCV on train (left) and test (right) set.

Table S1. Hyper-parameters set for each classifier.

Algorithms	Parameters
**Gaussian Naive Bayes**	priors=None
**Random Forest**	'max_depth': None, 'max_features': 'auto', 'min_samples_leaf': 1, 'min_samples_split': 2, 'n_estimators': 10, 'random_state': 90
**XGBoost**	'colsample_bytree': 1.0, 'learning_rate': 0.1, 'n_estimators': 10, 'subsample': 0.9
**KNeighborsClassifier**	'n_neighbors': 2, 'weights': 'uniform'
